# Cortical plasticity induced by different degrees of peripheral nerve injuries: a rat functional magnetic resonance imaging study under 9.4 Tesla

**DOI:** 10.1186/1749-7221-8-4

**Published:** 2013-05-09

**Authors:** Rupeng Li, Patrick C Hettinger, Jacques A Machol, Xiping Liu, J B Stephenson, Christopher P Pawela, Ji-Geng Yan, Hani S Matloub, James S Hyde

**Affiliations:** 1 Department of Biophysics, Medical College of Wisconsin, 8701 Watertown Plank Road, Milwaukee, WI 53226, USA; 2 Department of Plastic Surgery, Medical College of Wisconsin, Milwaukee, WI, USA; 3 Department of Anesthesiology, Medical College of Wisconsin, Milwaukee, WI, USA

**Keywords:** Functional magnetic resonance imaging (fMRI), Cortical plasticity, Peripheral nervous system (PNS), Central nervous system, Nerve injury, BOLD

## Abstract

**Background:**

Major peripheral nerve injuries not only result in local deficits but may also cause distal atrophy of target muscles or permanent loss of sensation. Likewise, these injuries have been shown to instigate long-lasting central cortical reorganization.

**Methods:**

Cortical plasticity changes induced after various types of major peripheral nerve injury using an electrical stimulation technique to the rat upper extremity and functional magnetic resonance imaging (fMRI) were examined. Studies were completed out immediately after injury (acute stage) and at two weeks (subacute stage) to evaluate time affect on plasticity.

**Results:**

After right-side median nerve transection, cortical representation of activation of the right-side ulnar nerve expanded intra-hemispherically into the cortical region that had been occupied by the median nerve representation After unilateral transection of both median and ulnar nerves, cortical representation of activation of the radial nerve on the same side of the body also demonstrated intra-hemispheric expansion. However, simultaneous electrical stimulation of the contralateral uninjured median and ulnar nerves resulted in a representation that had expanded both intra- and inter-hemispherically into the cortical region previously occupied by the two transected nerve representations.

**Conclusions:**

After major peripheral nerve injury, an adjacent nerve, with similar function to the injured nerve, may become significantly over-activated in the cortex when stimulated. This results in intra-hemispheric cortical expansion as the only component of cortical plasticity. When all nerves responsible for a certain function are injured, the same nerves on the contralateral side of the body are affected and become significantly over-activated during a task. Both intra- and inter-hemispheric cortical expansion exist, while the latter dominates cortical plasticity.

## Introduction

Nerve regeneration following peripheral nerve injury has been of interest to surgeons and neuroscientists for decades [1-5]. New surgical methods, such as partial nerve transfer and cross-C7 transfer, have been introduced to treat peripheral nerve injury [1,6-8]. Despite these advances in peripheral nerve surgery, functional recovery continues to have clinical shortcomings. A universal hypothesis examining this is that knowledge of the central nervous system’s (CNS) response post nerve injury and repair will lead to improved clinical procedures. This CNS plasticity following nerve injury and repair has been increasingly studied in recent years [9-15]. A recent study showed brain activation maps of the four major branches of the rat brachial plexus using functional magnetic resonance imaging (fMRI) [16]. Chen et al. [17] observed cortical rearrangement using fMRI following human toe-to-finger transplantation. Beaulieu et al. [6] reported that after cross-C7 grafts of the brachial plexus, cerebral plasticity could be observed using fMRI. They found that flexion of the neurotized arm is associated with bilateral cortical network activity. The contralateral cortex originally involved in control of the rescued arm still participates in the elaboration and control of the task through the bilateral premotor and primary motor cortices.

Functional MRI techniques are used in this investigation. These experiments are based on blood oxygen level dependent (BOLD) MRI contrast, which arises from change in oxygenation of hemoglobin that is associated with neural activity [18], noting that deoxyhemoglobin (dHb) is paramagnetic and oxyhemoglobin is diamagnetic [19]. A previous paper from our group reported detailed BOLD fMRI cortical representation maps of the rat that were obtained by stimulation of each of the four terminal branches of the brachial plexus [16].

Total deafferentation is the most severe case of peripheral nerve injury in the upper limb. The methodology of fMRI has been used to observe inter-hemispheric neuroplasticity following total forelimb deafferentation [20]. In another study, inter-hemispheric cortical plasticity deafferentation was also reported by Pelled et al. [15] following complete deafferentation.

In the present study, we report results obtained by transection in the rat forearm not only of a single nerve but also of nerve pairs. Given this, it was hypothesized that patterns of cortical plasticity caused by these types of peripheral nerve injuries would be different from those induced by total deafferentation. These differences could potentially lead to new and more refined rehabilitation and reeducation programs that may improve clinical outcomes.

**Figure 1:**
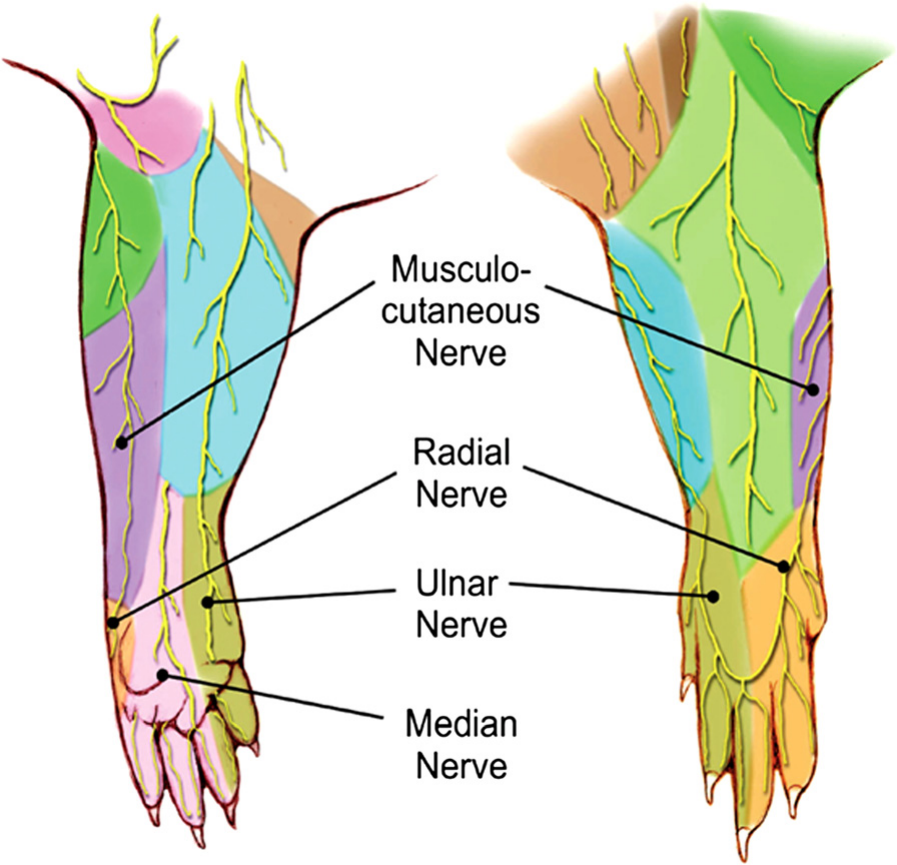
**Cutaneous innervation of the four terminal branches of the brachial plexus is shown.***Left*: volar. *Right*: dorsal.

Specifically, this study involves three major terminal branches of the brachial plexus in the rat upper extremity: the median, ulnar, and radial nerves (Figure 1). The radial nerve supplies sensory innervation to the dorsum of the forearm and dorsal aspect of the second, third, and a portion of the fourth digits. Other sensory input is conducted through median and ulnar nerve pairs. The median and ulnar nerves are responsible for flexion of the forearm and digits, while the radial nerve is responsible for extension. The fact that the median and ulnar nerves form a nerve pair with similar function allows us to study the different degrees of cortical plasticity arising from damage to one or both. This study uses surgical implantation of electrodes on the terminal branches of the rat brachial plexus for direct nerve stimulation [16,21]. This permits study of motor and sensory components of the cortical representations of the rat upper extremity.

## Materials and methods

### Animal preparation

**Figure 2:**
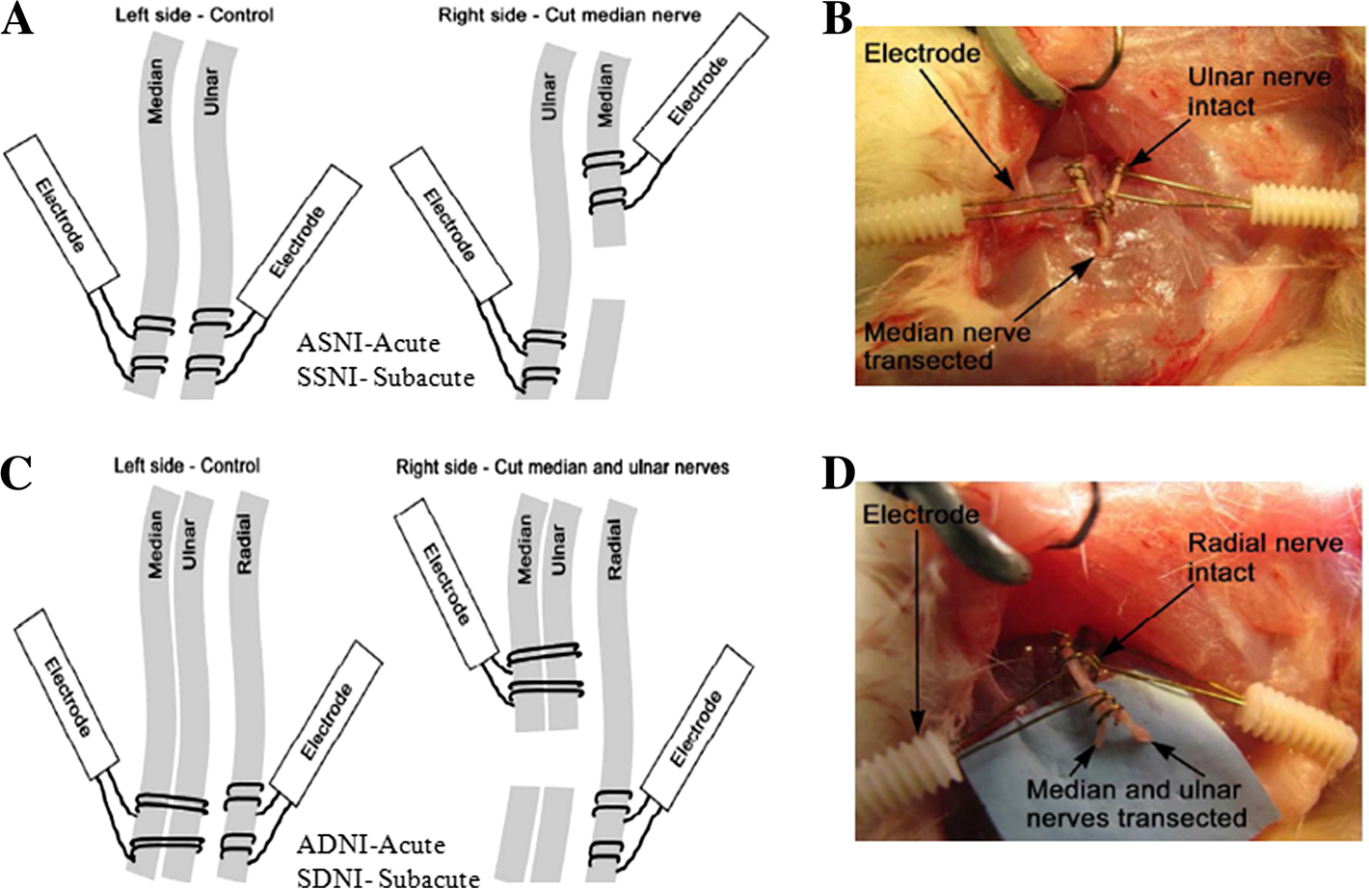
**The experimental design of the four groups is shown. **All nerve injuries were made to the right forelimb 1 cm proximal to the elbow. **A**: The experimental setup for rats in the ASNI and SSNI groups is illustrated. Here, the right median nerve was transected, and an electrode was placed on the proximal end of the transected nerve. A second electrode was placed on the ipsilateral right ulnar nerve. Third and fourth electrodes were placed on the contralateral left ulnar and median nerves, respectively. (These additional electrodes served as a control.) **C**: The experimental setup for rats in the ADNI and SDNI groups is shown. These rats had the right median and ulnar nerves transected, and electrodes placed on the proximal ends of the median and ulnar nerves. A second electrode was placed on the ipsilateral right radial nerve, a third was placed over the median and ulnar nerve bundle on the contralateral left side, and a fourth was placed on the contralateral left radial nerve. The left side also served as a control. **B **and **D **are intraoperative photos of the rat forelimb after electrodes were attached for the experimental setups in **A **and **C**, respectively. Note that the spiral electrode, designed in our laboratory, provides easy access to nerve stimulation with minimal damage to the nerve.

Institutional Review Board approval was obtained, and all studies were performed in compliance with federal regulations and the guidelines of our institution’s animal care and use committee. Twenty-four male Sprague–Dawley rats (150–200 g) were used in this study. They were divided equally into four groups based on the type of nerve injury and whether the injury was studied in the acute (30 minutes after injury) or subacute (two-week) stage of nerve injury. Animals that received only right median nerve transection and were examined in the acute stage constituted the “acute single nerve injury” (**ASNI**) group. Animals in the subacute single nerve injury (**SSNI**) group received the same median nerve injury but were examined in the subacute setting. Similarly, the double nerve injury groups—**ADNI** and **SDNI**—received right ulnar and median nerve transection followed by acute and subacute examinations, respectively. Schematic representations of the experimental setups for the ASNI and SSNI groups are shown in Figure 2A, with an intra-operative photo shown in Figure 2B. Similar schematic and intra-operative photos for the ADNI and SDNI groups are shown in Figure 2C and D, respectively.

In each rat, the brachial plexus was exposed bilaterally by an incision made along the medial aspect of the upper extremity and extending into the axilla and a portion of the flank. A plastic-sheet barrier was used to isolate the nerve, ensuring that the electrode fixed to the nerve contacted only the nerve to be stimulated. Stainless-steel, 150-μm-diameter bipolar electrodes (AISI 304, Plastics One, Roanoke, VA) were used in all experiments. All nerve injuries were made on the right side of the rat.

Rats in the ASNI and SSNI groups had the right median nerve transected 1 cm proximal to the elbow. Four electrodes were used in each rat: (i) the proximal end of the transected right median nerve, (ii) the right ulnar nerve, (iii) the left median nerve, and (iv) the left ulnar nerve. ASNI rats had electrodes placed at the time of injury, while SSNI rats were rested for two weeks followed by surgical implantation of electrodes just prior to the fMRI studies. All incisions were closed after surgery.

Rats in the ADNI and SDNI groups had the right median and ulnar nerves surgically transected 1 cm proximal to the elbow. Four electrodes were used in each rat: (i) the proximal ends of the transected right median and ulnar nerves bundled together, (ii) the right radial nerve, (iii) the left median and ulnar nerve bundle, and (iv) the left radial nerve. Electrodes were placed in ADNI rats at the time of injury. SDNI rats were rested for two weeks followed by surgical implantation of electrodes just prior to the fMRI studies. All incisions were closed after surgery.

Prior to fMRI acquisition, the right femoral artery and vein of each rat was cannulated for blood pressure monitoring and continuous intravenous (IV) drug administration. The trachea was cannulated to allow mechanical ventilation. The total surgical time, including nerve transection and placement of electrodes, varied from 1.5 to 2 hours. Rats in the ASNI and ADNI groups were scanned immediately after surgery to acquire data of cortical activation following acute nerve injury. Rats in the SSNI and SDNI groups underwent fMRI data acquisition after the second surgery to study the effects of subacute nerve injury.

### Anesthesia

Medetomidine hydrochloride (Domitor, Pfizer, New York, NY) has been used successfully in prior fMRI studies [22-24] and was used here. The rat was placed supine, and anesthesia was provided by 1% isoflurane vaporized into 30–70% O_2_/N_2_ during surgery. Isoflurane was tapered off during the fMRI portion of the study, and a continuous IV infusion of Domitor (Pfizer, USA. 0.1 mg/kg/hr) and pancuronium bromide ( Hospira, Lake Forest, IL. 2 mg/kg/hr) was used for maintenance of anesthesia and muscle paralysis. The rat was placed on a mechanical ventilator (MRI-1 Ventilator, CWE, Ardmore, PA) using 30–70% O_2_/N_2_.

### fMRI protocol

Each rat was placed on a custom-designed cradle fabricated from G-10 fiberglass material. The cradle was equipped with a warming pad controlled by a water-pump-driven temperature regulator (Medi-Therm III, Gaymar Industries, Orchard Park, NY).

Mild electrical stimulation at 10 Hz frequency, 0.5 mA current, and 1 ms duration was applied to the nerve trunk in all groups using a square-pulse electrical stimulator (S88 Square Pulse Stimulator, Grass Telefactor, West Warwick, RI) [16]. This stimulation intensity is non-noxious, and it does not require the participation of sensory bodies in the skin and cutaneous tissue that generate most of the painful sensation. It is the same technique that is used clinically to test nerve continuity intra-operatively on humans and has been shown to be innocuous [25]. We confirmed the non-noxious stimulation state by physiological monitoring. Throughout the stimulation session, there were no significant changes in arterial blood pressure, core temperature, or heart rate. Each nerve stimulation sequence began with an OFF period of 40 s followed by three repetitions of ON for 20 s and OFF for 40 s (total scan time = 3 min 40 s). This time sequence defines a reference waveform. The stimulation sequence was computer-controlled with LabVIEW software (National Instruments, Austin, TX) and started by a trigger pulse delivered by the scanner. Gradient echo (GE) scans (TE = 18.4 ms, TR = 2 s, matrix 96 × 96 and zero filled to 128 × 128, FOV = 4 cm, number of repetitions = 110, 10 contiguous 1 mm scans) were acquired using an echo-planar imaging (EPI) sequence on the 9.4 T/30 cm Bruker AVANCE MRI scanner (Bruker BioSpin, Billerica, MA) equipped with a receiving surface-coil (T9208) and a linear transmit-coil (T10325). Two sets of GE data were collected for each stimulation in order to ensure the reproducibility of our results. Each set of GE data consisted of a time course of images. In fMRI, a pixel time course waveform can be formed from the intensity of a given pixel in each image of an image time course. Cross-correlation of the reference waveform with every pixel time course was performed on each individual animal before group analysis. Maps of colorized correlation coefficients were overlaid on an anatomical image to produce an fMRI image. See Cho et al. [16] for more detail.

### Physiologic monitoring

During fMRI acquisition, invasive blood pressure, core body temperature, respiratory rate (Model 1025, SA Instruments, Stony Brook, NY), pulse oximetry (8600V, Nonin Medical, Plymouth, MN), arterial blood gases (i-Stat, Heska, Loveland, CO), and inspired/expired O_2_ and CO_2_ (POET IQ2, Criticare Systems, Waukesha, WI) were monitored (WinDaq Pro, DataQ Instruments, Akron, OH) and maintained within normal physiologic ranges. Rats were euthanized upon completion of the study.

### Data analysis

EPI scans were registered to ideal anatomy using the Oxford Center for Functional Magnetic Resonance Imaging of the Brain (FMRIB) Linear Image Registration Tool (FLIRT) software [26]. Data for each nerve and stimulation protocol were averaged and masked using Analysis of Functional NeuroImages (AFNI) software [27]. Activation was determined with a *P*-value threshold of 0.005 in group analysis after multiple comparison corrections (AlphaSim). Voxels were classified as active if the statistic was above threshold and counted (3dmaskave) across all slices. Voxel color-coding was determined by the amplitude of the fit coefficient. Regions of interest (ROIs) were drawn by consulting the activation map and cross-referencing it with the Paxinos and Watson rat atlas [28]. In this study, bilateral somatosensory cortex (S1) and motor cortex (M1) areas were selected as primary ROIs. With this method, the degree of cortical activation in different functional areas can be quantified by the area (or number) of activated voxels.

## Results

**Figure 3:**
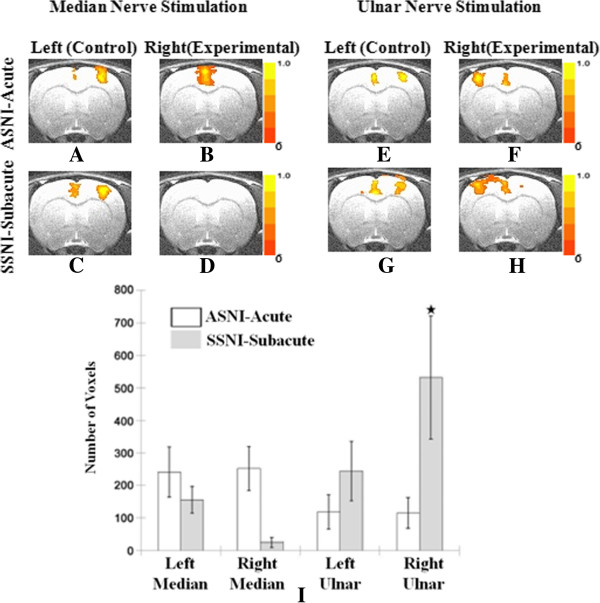
**Shown in Figure **3**A through H, cortical activation following nerve trunk stimulation in the ASNI and SSNI groups (P = 0.005). **Expansion was found in the ulnar nerve cortical representation, shown in Figure 3**F **and **H**. Figure 3**I **shows the statistical analysis of voxel-counting across slices. A significant change in voxel number between the acute and subacute stages was found in the representation of the right ulnar nerve (★ *P* < 0.05).

Figure 3 shows averaged results of coronal slices (-0.28 mm from bregma—the intersection between the coronal and sagittal sutures) from rats in the ASNI and SSNI groups. Figure 3A through *H* represent the cortical response during electrical stimulation of the specified nerve. Figure 3I shows an alternative means to quantify cortical activation by counting the number of activated voxels in the sensorimotor region of the cortex. The error bars in Figures 3I (and 4I) represent the standard deviation across animals. The right side of each coronal image shows the right cortex as by animal-imaging convention. The color scale depicts the intensity of the fMRI BOLD signal with the orange-yellow color range indicating a positive BOLD signal—a proxy for cortical activation. For example, Figure 3A shows cortical representation following stimulation of the left median nerve immediately after transection of the right median nerve. The midline positive BOLD region represents the motor and cingulate regions activated by median nerve stimulation, while the positive BOLD activation seen to the right of the midline represents the sensory region of the left median nerve. This is consistent with previous rat brain-mapping studies using direct nerve stimulation [16] Figure 3B is the cortical representation when the proximal end of the transected median nerve is stimulated. In this figure, the sensory region is no longer evident while the motor and cingulate regions, as well as some deeper structures of the basal ganglia, exhibit enhanced BOLD signals. This is a natural cortical response to acute injury and may be related to stress when animals are conscious or under light anesthesia [16,20,29-32]. This response to stimulation of the proximal end of the right median nerve is absent when the animal is examined in the subacute stage (Figure 3D).

**Figure 4:**
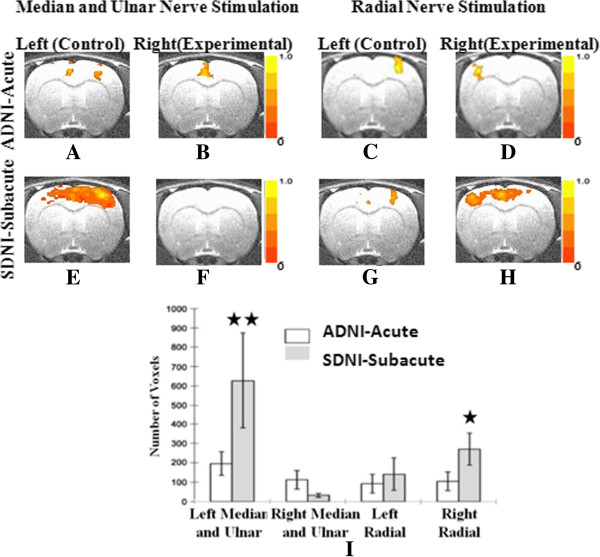
**Shown in Figure **3**A through H, cortical activation following nerve trunk stimulation in the ADNI and SDNI groups (*P*** **= 0.005).** Obvious expansion can be seen in the ulnar nerve cortical representation in Figure 4**A **and **C**. The right radial nerve also became overactivated in comparison to the healthy state, although this was not as prominent as the median and ulnar nerve bundle on the healthy side. The expansion not only became diffuse, but also entered the other hemisphere and occupied the motor area that previously represented the right median and ulnar nerve bundle. Figure 4I shows the statistical analysis of voxel-counting across slices. A significant change in voxel number between the acute and subacute stages was found in the representations of the left median and ulnar nerve bundles and the right radial nerve (★ *P* < 0.05; ★★ *P* < 0.01).

In the subacute stage, stimulation of the left median nerve continues to result in distinct localized sensorimotor activation (Figure 3C) that is similar to the activation observed in the acute stage (Figure 3A), with differences not of statistical significance (Figure 3I, left median). Figure 3E to H show cortical activation during the acute and subacute period arising from ulnar nerve stimulation on the right and left sides, respectively. There is no significant change in the activation area of the left-side ulnar nerve between acute and subacute stages (compare Figure 3E and G). The area of activation corresponding to the right ulnar nerve, however, increased when comparing the subacute to the acute stage (compare Figure 3F and H). This difference was shown to be statistically significant (Figure 3I). The expansion of the right ulnar nerve representation after right median nerve injury remained within the same hemisphere as the cortical representation of the right median nerve.

Figure 4 shows averaged results of coronal slices (-0.28 mm from bregma) for the ADNI and SDNI groups that underwent transection of the right median and ulnar nerve pair. Figures 4A to D show cortical activation in the acute and subacute injury stages when the median and ulnar nerve pairs on both sides were stimulated. In the acute stage, localized activation was found in the right sensorimotor cortex when stimulating the left nerve pair. In the subacute stage, stimulation of the left median and ulnar nerve pair showed an expanded area of cortical activation in the right sensorimotor area that also expanded into the left sensorimotor regions of the cortex (Figure 4C). The voxel count analysis shows this expansion to be statistically significant (Figure 4I, right median and ulnar). Cortical activation is absent in the subacute stage following stimulation of the injured right median and ulnar nerve pair (Figure 4D). Cortical activation in response to radial nerve stimulation is shown in Figure 4E through H. Cortical activation following stimulation of the left radial nerve remained essentially unchanged from the acute to subacute stages (Figure 4E and G). Stimulation of the right radial nerve resulted in a marked increase in sensorimotor activation in the left hemisphere and a moderate expansion into the motor regions of the right hemisphere. Stimulation of either the right radial nerve or the left median and ulnar nerve pair showed expansion of representation into the hemisphere contralateral to the stimulated side as well as into the ipsilateral hemisphere, which demonstrates inter-hemispheric cortical reorganization in response to nerve injury.

**Figure 5:**
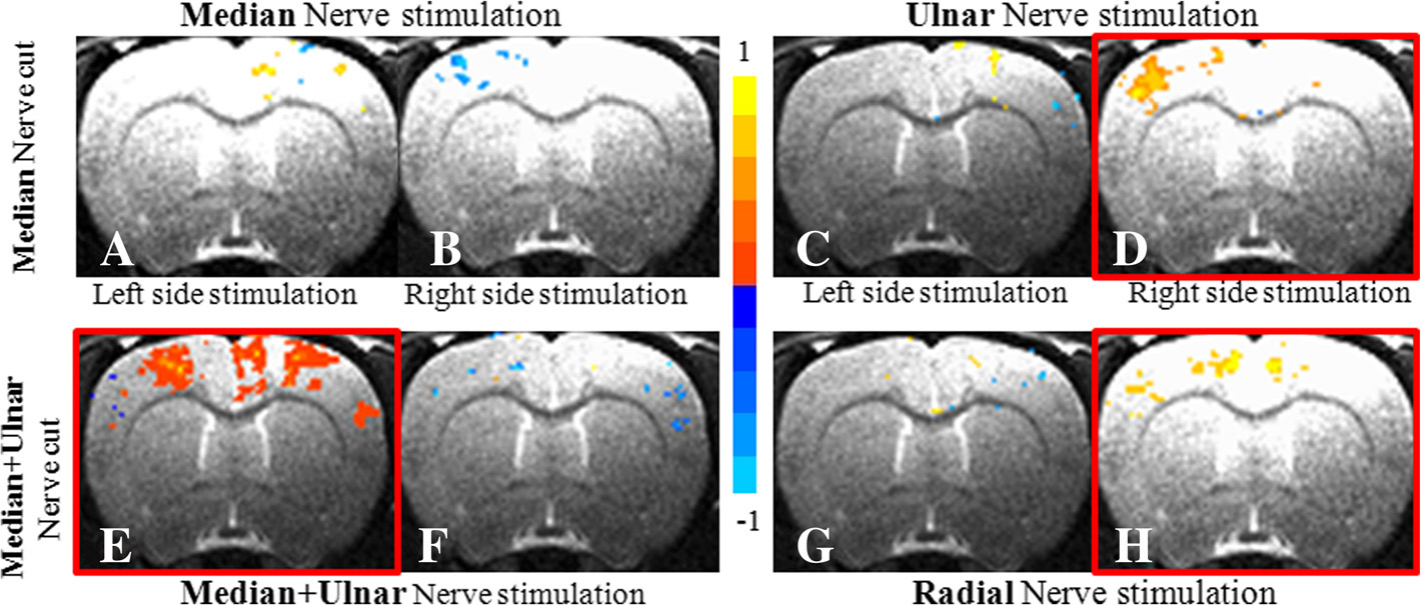
**Voxel-wise t-test showing amplitude change for all groups (p = 0.05). **All the analysis was done using acute stage of each group as baseline. **A**-**D** were from single median nerve injury groups. Significant intra-hemispheric cortical plasticity was demonstrated when right ulnar nerve was stimulated (**D**. circled in red). **E**-**H** were acquired from combined nerve injury group. Both inter-and intra-hemispheric plasticity were found during nerve stimulations, with the healthy nerve bundle on the healthy side being dominant (**E,H **circled in red).

In order to better demonstrate the cortical plasticity under different conditions, all fMRI signals from the sensorimotor cortex was extracted. Using the result from acute stage of each group as the baseline, voxel-wise t-test was performed at a p value of 0.05. Comparing to the voxel counting analysis in previous results, this analysis is able to show voxel-wised amplitude change. The result is demonstrated in Figure 5. In the single median nerve injury group (Figure 5A-D), all the plasticity happened intra-hemispherically. Despite the very localized and mixed changes when nerves from the healthy side were stimulated, cortical response was noticeably reduced when damaged median nerve was stimulated in the subacute stage (Figure 5B), shown in blue color. For this median nerve injury group, the most significant cortical plasticity occurred to the ulnar nerve of the same injury side (Figure 5D, circled by red). When compared to the acute stage, sensory activation was markedly increased in the amplitude. In the combined nerve injury group (Figure 5E-H), when median and ulnar nerve bundle on the healthy side and the radial nerve on the experimental side were stimulated, inter-hemispheric plasticity dominated the cortical changes (Figure 5E, H). The neuronal response also decreased when damaged nerves were simulated (Figure 5F). Only minimal changes could be detected when radial nerve on the healthy side was stimulated.

## Discussion

### Interaction between the CNS and PNS in nerve injury and repair

A well-functioning extremity requires not only a healthy peripheral nervous system including muscular and sensory end-organs, but also a healthy CNS. Peripheral nerve injuries remain one of the most challenging surgical problems and may result in devastating function loss that can have profound social consequences

Nerve injuries result not only in changes at the site of injury but also cause distal atrophy of target muscles or permanent loss of sensation. These injuries have been shown to cause long-lasting cortical reorganization [33,34]. Functional reorganization in the somatosensory and motor regions of the brain may explain the often disappointing results from severe peripheral nerve injuries and subsequent attempts at surgical repair. Clinically, there is no surgical repair technique that can assure full functional recovery following peripheral nerve repair.

Many studies have demonstrated that the functional outcome after nerve repair is superior in children than in adults [35,36]. Lundberg suggested that sensory recovery is based on a learning process that is analogous to learning a new language, which is a CNS function and is a type of cortical plasticity [37]. Based on these theories, many functional reeducation programs that focus on manipulating CNS plasticity have been used successfully in adult patients to improve functional outcomes following nerve repair. Sensory reeducation based on vision-guided touch [38] and constraint-induced therapy [39,40] have been shown to improve surgical outcome. In addition, pharmacological intervention studies have indicated that norepinephrine can improve functional outcome when combined with physical therapy [41,42].

As long as nerve repair is carried out with care and reasonable technical skill, the outcome appears to depend largely on CNS factors including functional cortical reorganization caused by misdirection in axonal outgrowth in the PNS [37]. Many studies suggest that the nature of cortical plasticity is to activate existing cortical pathways that are normally suppressed under healthy conditions [43-45]. Other studies insist that cortical plasticity is caused by newly formed axons [46,47]. Both may be correct. It is possible that different types of cortical plasticity. This is parallel to what was demonstrated in this study. We presume that both intra- and inter-hemispheric cortical expansion arise from activation of existing pathways since the actual functions of the median and ulnar nerves overlap and the left and right cortical representation areas for each nerve are connected across hemispheres by the corpus callosum. Our study established a reliable methodology in an animal model for visualization of cortical plasticity caused by nerve injury and/or repair, so that new intervention and reeducation procedures can be designed and evaluated.

When unrepaired nerve residue was stimulated, the neuronal activity in the corresponding cortex decreases markedly, resulting in a negative signal in voxel-wise t-test analysis (Figure 5B and F). Following cortical plasticity, the original cortical area of the damaged nerve seems to be partially taken by other nerves. This phenomenon has been well studied and documented in physiologic studies [9,48,49].

### Intra-hemispheric cortical plasticity

This study fills the gap the previous two studies left in which only inter-hemispheric plasticity was discussed[50,51]. In the present investigation, rapid changes were seen after injury. The sensory representation in the cortex upon stimulation of the proximal end of the cut nerve (compare Figure 3A and B) disappeared within two weeks (Figure 3D). More interesting are changes in the patterns of cortical activation that occur in response to stimulation of adjacent nerves on the same side of injury, as well as stimulation of contralateral nerves. The median nerve carries motor innervation that is primarily flexor in origin, with the balance of flexor innervation carried out by the ulnar nerve. When the median nerve is transected, the ipsilateral ulnar nerve representation in the cortex appears to expand during a two-week period to include more of the sensorimotor region. Apparently, the cortex reallocates resources to enhance sensorimotor interaction with the ulnar nerve in response to the loss of function resulting from injury to the median nerve. This finding is consistent with prior electrophysiology studies as well [34].

Intra-hemispheric cortical plasticity may occur because the resulting median nerve injury has not fully compromised flexor functioning of the forepaw digits and wrist. In addition, previous studies have demonstrated that there is overlap in cortical representation of the sensory components of the median, ulnar, and radial nerves [16]. Thus, with median nerve injury, it is not surprising for the ipsilateral ulnar nerve to exhibit expanded cortical representation (Figure 3H). This is further evidenced by studying the ADNI and SDNI groups. In these two collections, the left and right radial nerves were stimulated after injury to the right median and ulnar nerve pair (Figure 4E through H). In this setting, Intra-hemispheric cortical expansion was evident at two weeks upon stimulation of the right radial nerve (Figure 4H) but absent with stimulation of the left side radial nerve (Figure 4G). Voxel-wise t-test analysis for both single median nerve injury group and combined median+ulnar nerve injury group clearly demonstrates this intra-hemispheric cortical plasticity. It also substantiates that this increase in the neuronal activity during the subacute stage is caused by not only the increased neurons being fired during task, but also the increased intensity when each neuron fires.

### Inter-hemispheric cortical plasticity

The degree of functional loss was greater with transection of both the median and ulnar nerves compared to an isolated median nerve injury. Double nerve injury condition is fundamentally similar as previous published studies in which three and four major nerves of forelimb were destroyed in the sense that the entire biological function was knocked out. With this injury, the forepaw has only limited sensory and motor function, which is provided through the radial nerve. With the nerve pair injury used in this study, the involved limb completely loses the flexion function that is very essential to the daily movement of the animal. While in the previous studies, animals lost both flexion and extension functions as the result of complete limb deafferentation surgery. In order to deal with this complete function loss, appropriately, the cortical allocates resources to enhance interactions with the remaining uninjured ipsilateral radial nerve in order to maximize the remaining function of the injured extremity.

This observation of changing cortical representation reinforces the notion that any given region of the cortex may have multiple secondary synaptic connections beyond the primary innervation to that cortical region. These secondary connections are normally inhibited or masked in the non-injured state. In the setting of nerve injury, it is hypothesized that the primary innervation fails, resulting in an unmasking of these secondary connections and thus explaining the cortical expansion seen in representations of adjacent non-injured nerves [33].

Inter-hemispheric cortical expansion was observed in the setting of combined median and ulnar nerve injury, but absent with isolated median nerve injury. With median and ulnar nerve injuries, flexion of the forepaw digits and wrist is absent along with complete loss of volar sensation and a partial loss of dorsal sensation of the forepaw. With this degree of function loss, the rat becomes much more dependent on the uninjured forepaw compared to an isolated loss of median nerve function. Thus, stimulation of the left uninjured median and ulnar nerve pair displays significant intra- and inter-hemispheric cortical expansion. Inter-hemispheric cortical expansion implies that the two hemispheres are connected. However, stimulation of the left radial nerve displays neither intra- or inter-hemispheric cortical expansion, suggesting that the cortex does not need to enhance or devote more resources to the extensor function of the non-injured hand.

Only voxel-wise t-test analysis from the combined median and ulnar nerve injury group shows this inter-hemispheric cortical plasticity pattern (Figure 5E and H). Similar as it is in the single nerve injury group, for higher degree of nerve injury, both the neuron number and the intensity of neuronal response increase during the subacute stage. The difference is this over activation pattern involving both hemispheres.

What is clear from these two sets of experiments is that the gradation of functional loss produced by different degree of nerve injuries impacts the pattern of cortical reorganization. In addition, inter-hemispheric cortical expansion appears more likely to occur when there is a higher degree of functional loss in one extremity as the body and cortex try to response.

### Cortical expansion and cortical plasticity

Many studies have shown that reorganization of the rat cortex in response to peripheral nerve by either expansion of the representation of adjacent nerves on the same extremity or the representation of corresponding nerves on the side contralateral to nerve injury. This reorganization may be important when considering nerve repair options. It is not always possible for direct nerve repair or the use of cable grafts [52,53] to restore function to the denervated muscles or sensory end-organs. In these circumstances, a donor nerve may be an option, but choosing the appropriate donor can be challenging. A donor nerve on the same side as the nerve injury may exacerbate the degree of functional loss observed in the injured extremity. Choosing a contralateral donor nerve (such as the C7 nerve root) may be technically more challenging [7,54-56] and may require a longer period for the nerve-regeneration process. The emerging use of partial nerve transfers [57-59] may make sense in terms of impact on the cortical changes that occur in response to partial nerve redirection. The intra-hemispheric cortical expansion observed with stimulation of the ulnar nerve two weeks after median nerve injury suggests that a partial ulnar nerve transfer could be used to restore the relationship between the expanded cortical regions and reinnervated portions of the median nerve. Similarly, the implications of the intra-hemispheric cortical expansion observed with the right radial nerve stimulation two weeks after the right median and ulnar nerve injury suggests that a partial right radial donor nerve will help to restore the relationship between the expanded cortical region with some flexor function or protective volar sensation.

Both intra- and inter-hemispheric cortical expansion of the representations of the left median and ulnar nerves is observed two weeks after right nerve pair transection. It may be possible to use left median or ulnar nerve fascicles in a partial-nerve-transfer surgical strategy, which will help to reestablish the relationship with the expanded cortical regions and the motor and sensory regions of the reinnervated portions of the injured nerve pair. Similarly, stimulation of the left radial nerve two weeks after injury of the right median and ulnar nerve pair showed neither patterns of cortical expansion, suggesting that it may be more difficult to restore the cortical relationship with portions of the reinnervated right nerve pair when using a portion of the left radial nerve as a donor.

A possible concern about this study is that scar tissue around the nerve and electrodes could affect the efficiency of stimulation of the nerves in the subacute stage groups. Histology confirmed the presence of scarring in these groups. Scarring would be expected to reduce the effects reported here. Other uninjured nerves were not studied in these experiments, and the degree of cortical reorganization that might be observed in response to stimulation of these nerves could lead to additional candidate donors. Changes in the cortex in response to nerve injury and repair, including the use of different donor nerves, will need to be studied in future work.

## Conclusions

In conclusion, this study describes cortical plasticity in the sensorimotor cortex caused by peripheral nerve injury using direct nerve stimulation and fMRI. According to our study, when a nerve of the rat forelimb is injured, a nearby nerve with a similar function to the injured nerve becomes significantly over-activated. Under this condition, intra-hemispheric cortical expansion becomes the most significant component of cortical plasticity. When all nerves responsible for a certain function are injured, the same nerves on the contralateral side of the body are affected and become significantly over-activated during task. Both intra- and inter-hemispheric cortical expansion exist, while the latter dominates cortical plasticity.

## Competing interests

The authors declare that we have no competing interests. We declare that the interpretation of our data or the presentation of the comprehending information, are not influenced by a personal or financial relationship with other people or organizations. In the past five years we did not receive any reimbursements, fees, funding or salary from an organization that may in any way gain or lose financially from the publication of this manuscript, either now or in the future. We declare that we do not hold any stocks or shares in an organization that may in any way gain or lose financially from the publication of this manuscript, either now or in the future. We declare that we are not currently applying for any patents relating to the content of the manuscript. We declare that we do not have any other financial competing interests. Additionally, we declare that there are no non-financially competing interests (i.e. political, personal, religious, ideological, academic, intellectual, commercial or any other).

## Authors’ contributions

RL: has made contribution to experiment design, animal surgery and management, data collection & analysis, data interpretation, manuscript writing & critical revision. PCH: has made contribution to animal surgery & management and data collection. XL: has made contribution to experiment design, data collection & analysis, and manuscript revision. JBS: has made contribution to animal surgery, data collection and manuscript revision. CPP: has been involved to experiment design. JAM: has made contribution to experiment design and manuscript revision. JGY: has made contribution to experiment design and manuscript revision. HSM: has made contribution to experiment design, data interpretation and critical manuscript revision. JSH: PI of this study program, he has made contribution to experiment design, data collection interpretation, hardware support and critical manuscript revision. All authors have read and approved the final manuscript.
